# Multidetector computed tomography study to measure thoracic aorta diameters in Egyptian population

**DOI:** 10.1186/s43044-021-00216-y

**Published:** 2021-10-15

**Authors:** Ahmed Shehata Mohamed Ismail, Shareefa Ali Mohammad Al-Suraimi, Hossam El Din Ghanem El Hossary, Mohamed Ali Salem, Hossam Ibrahim Hamed Kandil

**Affiliations:** Cairo University, Giza, Egypt

**Keywords:** Thoracic aortic diameter, MSCT aortography, Aorta, Multi-slice CT, Aortography

## Abstract

**Background:**

The aorta is the largest and strongest artery in the body that plays an important role in the control of systemic vascular resistance and heart rate. Aortic diseases contribute to the wide spectrum of arterial diseases that may be diagnosed after a long period of subclinical development. Multidetector computed tomographic scanners (≥ 64 detector rows) for aortic imaging remain one of the most preferred imaging techniques for diagnosis and follow-up of aortic conditions in acute as well as chronic presentations. The aim of this study is to establish a normal reference values for aortic diameters among Egyptian population and to find which of the cardiovascular risk factors could be an independent determinant of the aortic diameters.

**Results:**

Five hundred and sixteen Egyptian individuals were enrolled in our study, the mean age was 53.5 ± 10.9, and males comprised 61.4% of the study population. Aortic root diameters measured at the annulus, sinus and STJ were 23.09 ± 2.55 mm, 33.75 ± 3.93 mm and 26.13 ± 3.05 mm, respectively. The BSA-indexed diameters were 11.70 ± 1.39, 17.10 ± 2.10 and 13.25 ± 1.65, respectively. The diameter of the tubular part of ascending aorta was 30.97 ± 4.16 mm, and the BSA-indexed diameter was 15.71 ± 2.28. The aortic diameters measured at the level of the pulmonary bifurcation were 24.56 ± 2.95 mm and 23.79 ± 2.96 mm at systolic and diastolic phases, respectively. The BSA-indexed diameters were 12.44 ± 1.52 and 12.05 ± 1.52 at systolic and diastolic phases, respectively. At the diaphragmatic level, the mean diameters were 22.39 ± 2.72 mm and 21.49 ± 2.79 mm at systolic and diastolic phases, respectively. The BSA-indexed diameters were 11.34 ± 1.43 and 10.98 ± 1.48 at systolic and diastolic phases, respectively. Age, gender, BSA, BMI and hypertension were statistically significant independent predictors of ascending and descending aortic diameters.

**Conclusions:**

Our study established a normal reference value for thoracic aortic diameters among Egyptians using contrast enhanced MSCT aortography. Age, Gender, BSA, BMI and hypertension are the major determinants of aortic diameters.

## Background

The aorta is the largest and strongest artery in the body; its wall consists of three layers: the thin inner layer or intima, a thick middle layer or media, and a rather thin outer layer, or adventitia. The aorta is also an ultimate conduit, carrying, in average lifetimes, almost 200 million liters of blood to the body. In addition to the conduit function, the aorta plays an important role in the control of systemic vascular resistance and heart rate, via pressure-responsive receptors located in the ascending aorta and the aortic arch [1].

Aortic diseases contribute to the wide spectrum of arterial diseases: aortic aneurysms, acute aortic syndromes (AAS) and so many others. Similarly, to other arterial diseases, aortic diseases may be diagnosed after a long period of subclinical development [1].

There have been remarkable advances in noninvasive imaging of aortic diseases using echocardiography, CT and MRI. Because of these advances, acquiring more accurate measurements for the aorta and establishing the major anthropometric and clinical determinants have become more accessible and knowing the normal aortic diameters and the physiological variations has become essential for rapid diagnosis and treatment or even for screening purposes in order to lower fatalities resulting from aortic diseases [2].

Multidetector computed tomographic scanners (≥ 64 detector rows) for aortic imaging remain one of the most frequently used and preferred imaging techniques for diagnosis and follow-up of aortic conditions in acute as well as chronic presentations. Its advantages over other imaging modalities include the short time required for image acquisition and processing, the ability to obtain a complete 3D dataset of the entire aorta, and its accuracy and reproducibility, as well as its speed, simplicity, and widespread availability.

In this study, we will try to establish a normal reference values for aortic diameters among Egyptian population using contrast-enhanced ECG-gated MDCT, and to find which of the cardiovascular risk factors could be an independent determinant of the aortic diameters.

## Methods

*Study design and population* This is a non-randomized, observational, cross-sectional study that enrolled 516 Egyptian patients (199 females, 317 males) who came for MSCT coronary angiography.

### Consent for publication

All patients included in this research gave written informed consent to publish the data contained within this study.

*Inclusion criteria* Individuals > 20 years old who were scheduled for MSCT coronary angiography.

## Exclusion criteria

1. Known aortic valve disease.
2. Known aortic artery disease.
3. Previous CABG.
4. Individuals who refuse to participate in this study.

### Study design

1. Clinical assessment: All individuals were subjected to detailed medical history and clinical examination with special emphasis on:
  - Demographic characteristics and anthropometric measurements:
    Age and gender
    Height measurement using the standing height scale
    Body weight and Body mass index (BMI was calculated using the following formula: BMI = body weight (kg)/height (m^2^). Classification of adults according to BMI (table adapted from WHO Consultation on Obesity. Geneva, 1997) [3]. Classification according to BMI (kg/m^2^): Underweight < 18.5 Normal range 18.5–24.9 Overweight 25–29.9 Obese class I 30–34.9 Obese class II 35–39.9 Obese class III ≥ 40
    Body surface area (BSA) was calculated using Du Bois formula: BSA = 0.007184 × W0.425 × H0.725.
  - Cardiovascular risk factors: DM, hypertension, dyslipidemia, smoking and CAD.
2. Multi-slice CT Angiogram (MSCT) Study Protocol: Multi-slice CT coronary angiography was performed using the ECG-gated acquisition during a single breath hold by 320-slice, Toshiba Aquilion one machine (Kern: FC43, spacing 0.25 mm, FOV 240 mm, thickness 0.5 mm, 120 kV, 450 mA). Injection of 75 ml of non-ionic contrast material was administered through an anti-cubital vein at a high flow rate (5.3 ml/sec.) followed by rapid acquisition of constructive ultra-thin sections through the heart and its great vessels to evaluate the coronary arteries and the thoracic aorta. The study was evaluated at 75% and 40% of the cardiac cycle with selective reconstruction of the improperly visualized segments at different phases of the cardiac cycle. Dedicated software: Aquarius iNtuition edition Version 4.4.6 TeraRecon INC was used to analyze the data. Aortic diameters were measured at the following intra-thoracic levels:
  Aortic annulus.
  Aortic valve sinus.
  Sinotubular junction.
  Tubular part of the ascending aorta at its maximum dimension.
  Aorta at the levels of pulmonary bifurcation and diaphragm.

Diameters were measured at the systolic (40%) and diastolic (75%) phases of the cardiac cycle, and then the mean of the systolic and diastolic diameters were calculated.

**Fig. 1:**
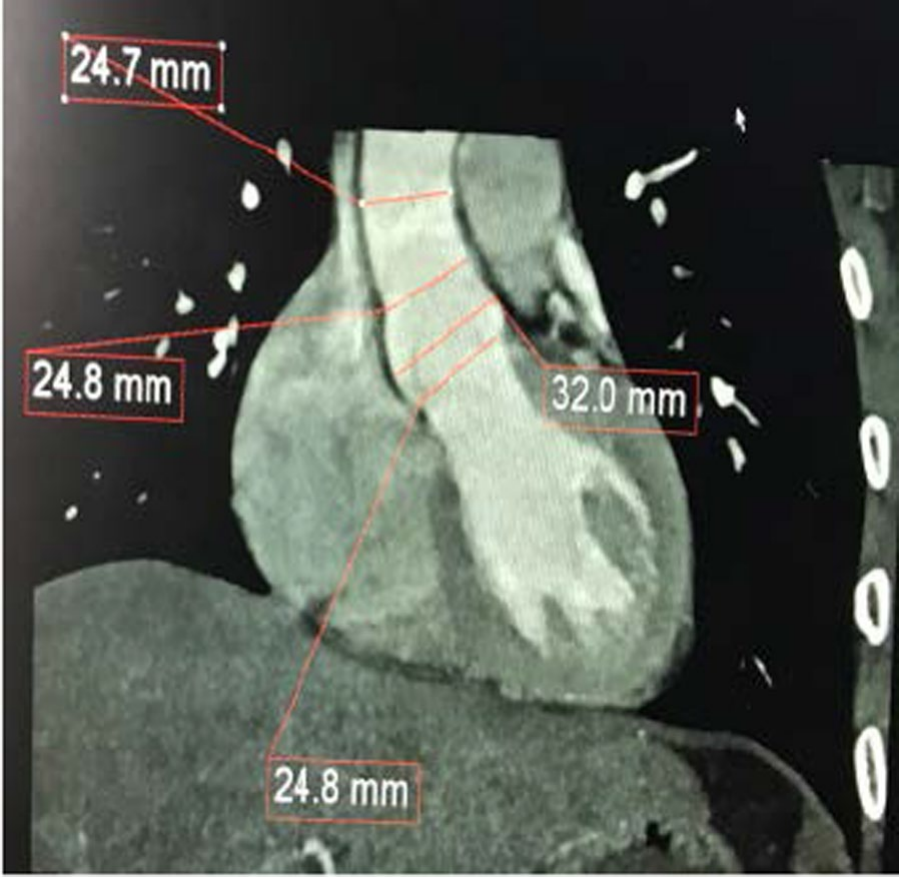
ECG-gated cardiac computed tomography (coronal view) in a healthy 32-year-old man

**Fig. 2:**
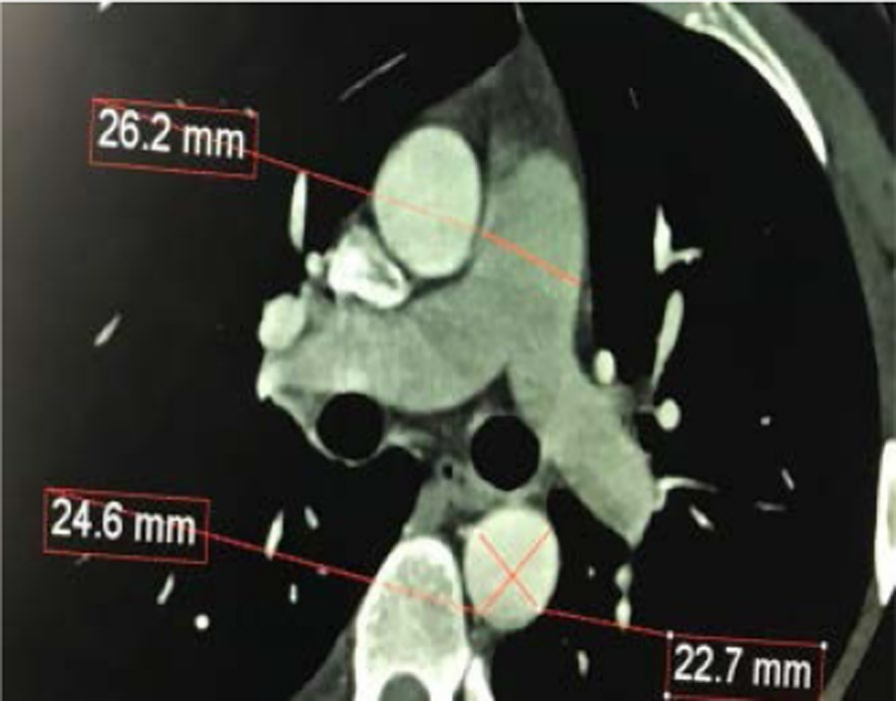
ECG-gated cardiac computed tomography (axial view) of the descending thoracic aorta and the pulmonary artery in a healthy 32-year-old man

**Fig. 3:**
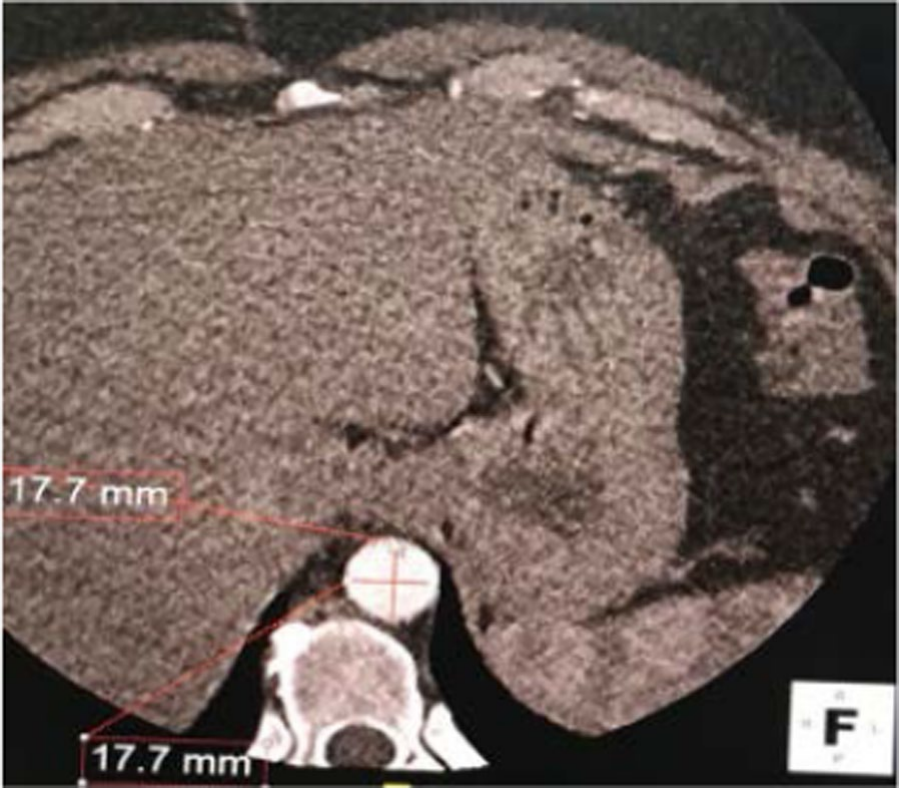
ECG-gated cardiac computed tomography (axial view) of the descending thoracic aorta at the level of the diaphragm in a healthy 38-year-old woman

Measurements of the aortic annulus, aortic sinus and STJ were taken hinge to hinge distance (from the outer upper side to the inner lower side) on coronal view (Fig. 1). Axial measurements (long and short diameters) of the aorta at the levels of the pulmonary bifurcation (Fig. 2) and diaphragm were measured (Fig. 3).

Coronary artery disease severity was assessed using the SCCT Grading scale; mild stenosis: 25–49%; moderate: 50–69%; severe: 70–99% [4]. Coronary ectasia is defined as dilatation with a diameter of 1.5 times the adjacent normal coronary artery [5].

*Primary outcome* To establish normal reference values of aortic dimensions among Egyptian population.

*Secondary outcome* To establish the major determinants of thoracic aortic diameter at its different levels.

### Statistical methodology

After data were collected, they were analyzed by Statistical Package of Social Science (SPSS) version 19. Categorical data are described as number and percentages, and continuous data are described as means ± SD (for normally distributed data) or median and range for abnormally distributed data. A Student’s t test (for data that was normally distributed) or a Mann–Whitney test (for data that was not normally distributed) and Pearson Chi-square test (for data that were categorical variables) were used for conducting comparison analysis. Linear regression analysis was used to predicted aortic diameters at its different levels. Two-tailed *P* value < 0.05 was considered significant.

## Results

This non-randomized, observational, cross-sectional study was carried out in Cairo, Egypt, from November 2017 to November 2018. This study enrolled 516 Egyptian patients who underwent CT coronary angiography for evaluation of coronary artery disease.

### Demographic and baseline clinical characteristics of the study population

Five hundred and sixteen Egyptian individuals were enrolled in our study, the mean age was 53.5 ± 10.9, males comprised 61.4% of the study population, the mean weight and height were 88.3 ± 15.5 kg and 169.4 ± 9.7 cm, respectively, the mean BMI was 30.9 ± 5.5 cm^2^/kg, and the mean BSA was 1.984 ± 0.193 m^2^. Most of the patients were overweight and mildly obese 67.3%. These data are present in Tables 1 and 2.

**Table 1:** Baseline demographic and anthropometric measures of the study population

Variables	Mean ± SD
Age (years)	53.5 ± 10.9
Gender, male	317 (61.4%)
Height (cm)	169.4 ± 9.7
Weight(kg)	88.3 ± 15.5
BMI (cm^2^/kg)	30.9 ± 5.5
BSA(m^2^)	1.984 ± 0.193

**Table 2:** Obesity distribution among the study population

BMI category	Number	Percent %
Normal (18.5–24.9)	60	11.6
Overweight (25–29.9)	180	34.9
Obesity Class I (30–34.9)	167	32.4
Obesity Class II (35–39.9)	73	14.1
Obesity Class III (≥ 40)	36	7.0

Most of the patients were hypertensive and dyslipedemic. About half of the patients had CAD. Minority was smokers and diabetic. The prevalence of cardiovascular risk factors presented in Table 3.

**Table 3:** Baseline clinical characteristics of the study population

Variables	Count %
HTN	305 (59.1%)
DM	124 (24%)
Smoker	166 (32.2%)
Dyslipidemia	305 (59.1%)
CAD	240 (46.5%)

	Mean ± SD
SBP (mmHg)	131.3 ± 17.7
DBP (mmHg)	79.7 ± 10.5
Ca score	43.6 ± 150.9

HTN: hypertension, DM: diabetes mellitus, CAD: coronary artery disease, SBP: systolic blood pressure, DBP: diastolic blood pressure

We divided the study population into 3 groups according to their age; group A, B and C, both (Table 4).

**Table 4:** Age groups

Variables	Number (%)
Group A < 40 y	69 (13.4%)
Group B 40–60 y	312 (60.5%)
Group C > 60 y	135 (26.2%)

### Aortic diameter measurements

#### Ascending aorta

Aortic root diameters measured at the annulus, sinus and STJ were 23.09 ± 2.55 mm, 33.75 ± 3.93 mm, 26.13 ± 3.05 mm, respectively. The BSA-indexed diameters were 11.70 ± 1.39, 17.10 ± 2.10, 13.25 ± 1.65, respectively. The diameter of the tubular part of ascending aorta was 30.97 ± 4.16 mm, and the BSA-indexed diameter was 15.71 ± 2.28 (Table 5).

**Table 5:** Diameters of the ascending aorta at its root and tubular part

Variable	Mean ± SD (mm)
*Annulus diameter*	
Systole	24.24 ± 2.70
Diastole	21.95 ± 2.76
Mean (systole and diastole)	23.09 ± 2.55
Indexed	11.70 ± 1.39
*Sinus diameter*	
Systole	33.91 ± 4.01
Diastole	33.60 ± 3.90
Mean (systole and diastole)	33.75 ± 3.93
Indexed	17.10 ± 2.10
*STJ diameter*	
Systole	26.32 ± 3.12
Diastole	25.95 ± 3.10
Mean (systole and diastole)	26.13 ± 3.05
Indexed	13.25 ± 1.65
*Tubular diameter*	
Systole	31.61 ± 4.19
Diastole	30.34 ± 4.24
Mean (systole and diastole)	30.97 ± 4.16
Indexed	15.71 ± 2.28

#### The descending aorta

The aortic diameters measured at the level of the pulmonary bifurcation were 24.56 ± 2.95 mm and 23.79 ± 2.96 mm at systolic and diastolic phases, respectively. The BSA-indexed diameters were 12.44 ± 1.52 and 12.05 ± 1.52 at systolic and diastolic phases, respectively (Table 6). At the diaphragmatic level, the mean diameters were 22.39 ± 2.72 mm and 21.49 ± 2.79 mm at systolic and diastolic phases, respectively. The BSA-indexed diameters were 11.34 ± 1.43 and 10.98 ± 1.48 at systolic and diastolic phases, respectively (Table 7).

**Table 6:** Diameters of descending aorta at pulmonary bifurcation level

Variable	Mean ± SD (mm)
*Descending aorta at pulmonary bifurcation level*	
Mean of systolic and diastolic diameters	24.17 ± 2.92
Indexed diameters of the mean systolic and diastolic diameters	12.24 ± 1.51

**Table 7:** Diameters of descending aorta at diaphragmatic level

Variable	Mean ± SD (mm)
*Descending aorta at diaphragmatic level*	
Mean of systolic and diastolic diameters	21.94 ± 2.73
Indexed mean systolic and diastolic diameters	11.11 ± 1.44

### The coronary arteries

We examined the coronary arteries and found that 276 (53%) of the study population had normal coronaries and the rest had mild to severe coronary stenosis (Table 8). We adapted the SCCT Grading scale for coronary stenosis severity. Cury.et al. [4]. About 14.53 % of the study population had coronary ectasia.

**Table 8:** Coronary artery stenosis severity

Variable	Number and (%)
*Coronary artery stenosis severity*	
Mild	73 (41.1)
Moderate	68 (13.2)
Severe	99 (19.2)

Stenosis; Mild: 25–49%; Moderate: 50–69%; Severe: 70–99%; Cury et al. [4]

### Comparison analysis

#### Anthropometric and clinical characteristics in different age groups

We compared the anthropometric characteristics in the different age groups. There was a statistically significant difference between the groups regarding height, BMI and BSA. Older groups (> 40 y) had larger BMI and smaller BSA than those younger than 40 years old (Table 9).

**Table 9:** Comparison analysis of anthropometric measurements among different age groups

Variables	Age groups			*P* value
	Group A (< 40 y) (*n* = 69)	Group B (40–60 y) (*n* = 312)	Group C (> 60 y) (*n* = 135)	
	Mean ± SD			
Height (cm)	173 ± 8.1	169 ± 9.8	166.3 ± 9.3	**< 0.001**
Weight (kg)	86.4 ± 15.7	89.6 ± 16.0	86.2 ± 14.1	0.065
BMI (cm^2^/kg)	28.9 ± 5.4	31.1 ± 5.4	31.3 ± 5.6	**0.001**
BSA (m^2^)	1.995 ± 0.178	2.001 ± 0.202	1.939 ± 0.171	**0.012**

Bold mean significant values that is less than or equal 0.05

**Fig. 4:**
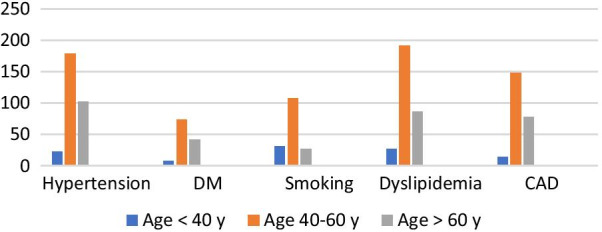
Comparison of clinical characteristics among different age groups

We compared the different age groups regarding their gender and cardiovascular risk factors. There was a statistically significant difference among the groups regarding gender, cardiovascular risk factors and SBP. Older groups (> 40 years old) had higher burden of cardiovascular risk factors (Table 10, Fig. 4).

**Table 10:** Comparison of demographic and clinical characteristics among different age groups

Variable	Age groups			*P* value
	Group A (< 40 y) (*n* = 69)	Group B (40–60 y) (*n* = 312)	Group C (> 60) (*n* = 135)	
	Number (%)			
Gender, male	52 (75%)	199 (63%)	66 (48%)	**< 0.001**
HTN	23 (33%)	179 (57%)	103 (76%)	**< 0.001**
DM	8 (11%)	74 (23%)	42 (31%)	**0.008**
Smoking	31 (44.9%)	108 (34%)	27 (20%)	**0.001**
Dyslipidemia	27 (39%)	192 (61%)	86 (63%)	**0.001**
CAD	14 (20%)	148 (47%)	78 (57%)	**< 0.001**

		Mean ± SD		
SBP mmHg)	123.0 ± 16.0	130.1 ± 16.7	138.3 ± 9.3	**< 0.001**
DBP (mmHg)	78.8 ± 11.9	79.7 ± 10.1	80.3 ± 10.6	0.624

Bold mean significant values that is less than or equal 0.05

### Aortic diameters in different age groups

**Fig. 5:**
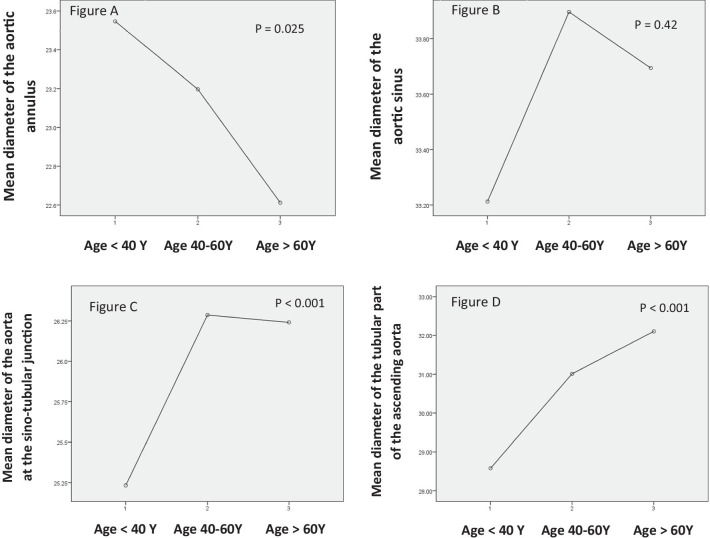
**a** Mean diameter of the aortic annulus in the age groups (< 40 Y, 40–60 Y and > 60 Y), **b** mean diameter of the aortic sinus in the age groups (< 40 Y, 40–60 Y and > 60 Y), **c** mean diameter of the aortic sino-tubular junction in the age groups (< 40 Y, 40–60 Y and > 60 Y), **d** mean diameter of the tubular part of the ascending aorta in the age groups (< 40 Y, 40–60 Y and > 60 Y)

We compared aortic diameters at its different levels among the different age groups. There was a statistically significant difference in the aortic diameters measures at the annulus, sinus, STJ and tubular part among the different age groups. While aortic diameters measured at the level of the sinus, STJ and the tubular part appear to larger with increasing age, the aortic annulus appears smaller in older groups (Table 11, Fig. 5).

**Table 11:** Comparison of ascending aortic diameters (root and tubular parts) among different age groups

Diameter (mm)	Age group			*P* value
	Group A (< 40 y) (*n* = 69)	Group B (40–60 y) (*n* = 312)	Group C (> 60 y) (*n* = 135)	
	Mean ± SD			
*Annulus*				
Mean	23.55 ± 2.54	23.20 ± 2.62	22.61 ± 2.34	**0.025**
Indexed	11.86 ± 1.39	11.66 ± 1.44	11.71 ± 1.26	0.456
*Sinus*				
Mean	33.21 ± 3.66	33.90 ± 4.08	33.69 ± 3.70	0.426
Indexed	16.72 ± 1.93	17.03 ± 2.13	17.46 ± 2.08	**0.029**
*STJ*				
Mean	25.23 ± 2.51	26.29 ± 3.19	26.24 ± 2.90	0.057
Indexed	12.71 ± 1.40	13.21 ± 1.70	13.59 ± 1.58	**< 0.001**
*Tubular*				
Mean	28.58 ± 3.88	31.01 ± 3.94	32.10 ± 4.31	**< 0.001**
Indexed	14.39 ± 2.04	15.59 ± 2.08	16.6 ± 2.46	**< 0.001**

Bold mean significant values that is less than or equal 0.05

**Fig. 6:**
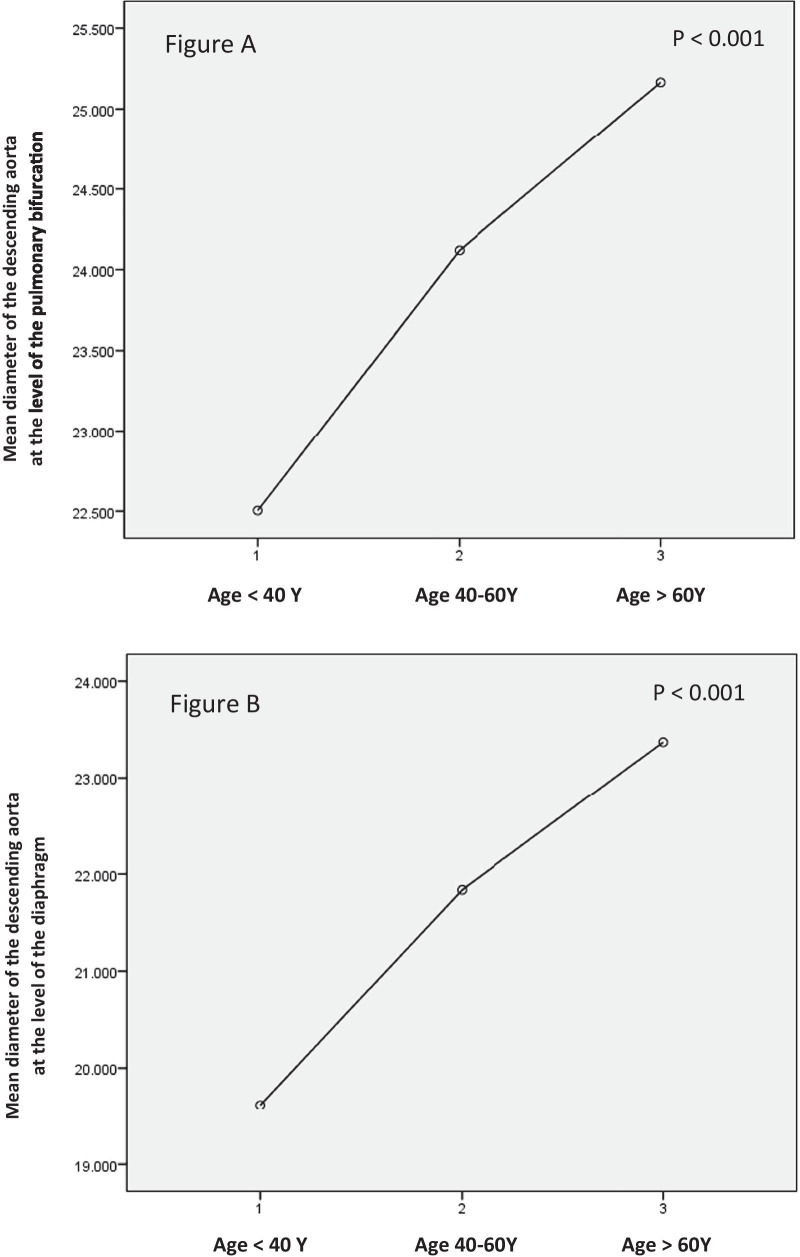
**a** Mean diameters of the descending aorta at the pulmonary bifurcation level in the age groups (< 40 Y, 40–60 Y and > 60 Y) and **b** mean diameters of the descending aorta at the diaphragm in the age groups (< 40 Y, 40–60 Y and > 60 Y)

There was a statistically significant difference in all descending thoracic aorta diameters (mean and BSA-indexed) at the level of pulmonary bifurcation. Aortic diameters increased with age (Table 12, Fig. 6).

**Table 12:** Comparison of descending thoracic aortic diameters at pulmonary bifurcation level among different age groups

Variable (Diameter)	Age groups				*P* value
		Group A (< 40 y) (*n* = 69)	Group B (40–60 y) (*n* = 312)	Group C (> 60 y) (*n* = 135)	
	Mean ± SD (mm)				
Descending aorta at pulmonary bifurcation	Mean of systolic and diastolic diameters	22.5 ± 2.2	24.1 ± 2.8	25.1 ± 2.9	< 0.001
	Indexed mean systolic and diastolic diameters	11.3 ± 1.1	12.1 ± 1.4	13.0 ± 1.4	< 0.001

Likewise, there was a statistically significant difference in the descending thoracic aorta diameters (mean and BSA-indexed) at the diaphragmatic level. Aortic diameters increased with age. (Table 13)

**Table 13:** Comparison of descending thoracic aortic diameters at the diaphragmatic level among different age groups:

Variable (diameter)	Age group				*P* value
		Group A (< 40 y) (*n* = 69)	Group B (40–60 y) (*n* = 312)	Group C (> 60 y) (*n* = 135)	
	Mean ± SD (mm)				
Descending thoracic aorta at diaphragm	Mean of systolic and diastolic diameters	19.6 ± 1.8	21.8 ± 2.4	23.3 ± 2.8	< 0.001
	Indexed mean systolic and diastolic diameters	9.8 ± 1.0	10.9 ± 1.2	12.0 ± 1.3	< 0.001

### Anthropometric and clinical characteristics according to gender

When comparing the different anthropometric characteristics according to gender, males were taller, heavier with larger both body mass index and body surface area and higher blood pressure. Females were older (Table 14)

**Table 14:** Comparison of anthropometric and clinical characteristics of the study population according to gender

Variable	Gender		*P* value
	Female (*n* = 199)	Male (*n* = 317)	
	Mean ± SD		
Age	56.39 ± 10.70	51.62 ± 10.62	**< 0.001**
Height	161.6 ± 8.3	174.2 ± 6.9	**< 0.001**
Weight	83.5 ± 15.1	91.2 ± 15.0	**< 0.001**
BMI	32.1 ± 6.3	30.1 ± 4.8	**0.001**
BSA	1.871 ± 0.173	2.056 ± 0.170	**< 0.001**
SPB	132.8 ± 19.4	130.3 ± 16.5	0.175
DPB	78.0 ± 10.3	80.8 ± 10.5	**0.002**

Bold mean significant values that is less than or equal 0.05

### Aortic diameters according to gender

Table 15 shows that aortic annulus diameter, sinus diameter and the STJ diameter were statistically significant, larger in males. Regarding the tubular aortic diameter, there was statistically significant difference in the BSA-indexed diameter between men and women.

**Table 15:** Comparison of ascending aortic diameters according to gender

Variable	Female	Male	*P* value
	Mean ± SD	Mean ± SD	
*Annulus diameter*			
(Mean)	21.48 ± 2.03	24.10 ± 2.32	**< 0.001**
(BSA-indexed)	11.56 ± 1.44	11.79 ± 1.34	**0.030**
*Sinus diameter*			
(Mean)	31.08 ± 3.06	35.43 ± 3.46	**< 0.001**
(BSA-indexed)	16.75 ± 2.24	17.32 ± 1.98	**< 0.001**
*STJ diameter*			
(Mean)	24.65 ± 2.47	27.06 ± 3.01	**< 0.001**
(BSA-indexed)	13.27 ± 1.68	13.23 ± 1.63	0.840
*Tubular diameter*			
(Mean)	30.55 ± 4.00	31.24 ± 4.24	0.091
(BSA-indexed)	16.43 ± 2.31	15.26 ± 2.15	**< 0.001**

Bold mean significant values that is less than or equal 0.05

**Fig. 7:**
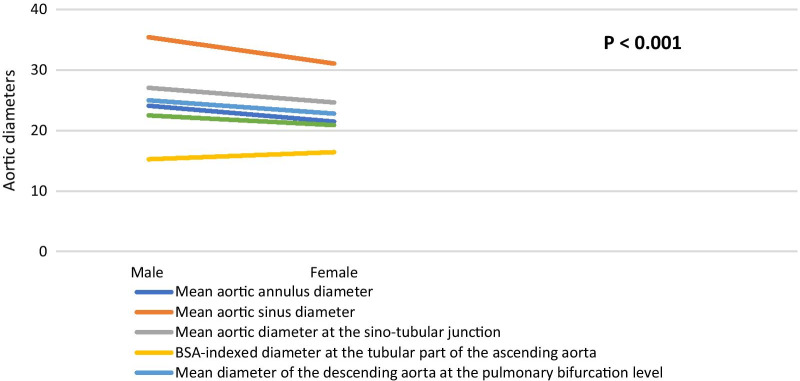
Diameters of the ascending and descending aorta at their different levels in male and female gender

We compared the descending aortic diameters between males and females at the levels of pulmonary bifurcation and diaphragm. There was a statistically significant difference in all mean diameters. They were larger in men than women. BSA-indexed diameters were larger in women; however, they were not statistically significant (Table 16, Fig. 7).

**Table 16:** Comparison of descending aortic diameters according to gender

Variable	Female	Male	*P* value
	Mean ± SD	Mean ± SD	
*Descending aorta at diaphragm*			
Mean of systolic and diastolic diameters	20.9 ± 2.4	22.5 ± 2.7	**< 0.001**
Indexed mean systolic and diastolic diameters	11.2 ± 1.3	11.0 ± 1.4	0.11
*Descending aorta at pulmonary bifurcation*			
Mean of systolic and diastolic diameters	22.8 ± 2.6	25.0 ± 2.7	**< 0.001**
Indexed mean systolic and diastolic diameters	12.2 ± 1.5	12.2 ± 1.4	0.59

Bold mean significant values that is less than or equal 0.05

### Aortic diameter and hypertension

**Fig. 8:**
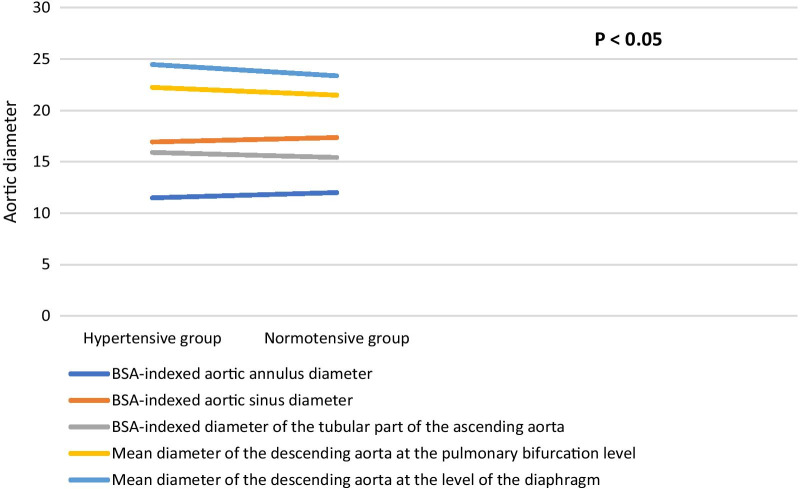
Comparison of aortic diameters at its different levels between hypertensive and normotensive groups

Comparing ascending aorta diameters in hypertensive and non-hypertensive patients (Table 17, Fig. 8) showed that indexed diameters at the levels of the annulus, aortic sinus and the tubular part are statistically significant larger in hypertensive patients. There was no statistical significant difference in the sino-tubular junction diameter in both groups.

**Table 17:** Comparison of ascending thoracic aorta diameters in normotensive and hypertensive individuals

Variable	Normotensive	Hypertensive	*P* value
	Mean ± SD	Mean ± SD	
*Annulus diameter*			
(Mean)	23.33 ± 2.66	22.92 ± 2.46	0.175
(BSA-indexed)	12.00 ± 1.42	11.49 ± 1.32	**< 0.001**
*Sinus diameter*			
(Mean)	33.74 ± 4.07	33.76 ± 3.83	0.803
(BSA-indexed)	17.36 ± 2.20	16.93 ± 2.01	**0.037**
*STJ diameter*			
(Mean)	25.99 ± 3.00	26.23 ± 3.09	0.452
(BSA-indexed)	13.38 ± 1.69	13.15 ± 1.62	0.176
*Tubular diameter*			
(Mean)	29.94 ± 3.94	31.69 ± 4.17	**< 0.001**
(BSA-indexed)	15.42 ± 2.20	15.91 ± 2.32	**0.015**

Bold mean significant values that is less than or equal 0.05

Comparing the descending aorta at the level aortic bifurcation or the level of the diaphragm (Table 18) showed that hypertensive patients had statistically significant larger mean diameters.

**Table 18:** Comparison of descending thoracic aorta diameters in normotensive and hypertensive individuals

Variable diameter (mm)	Normotensive	Hypertensive	*P* value
	Mean ± SD	Mean ± SD	
*Descending aorta at the diaphragm*			
Mean of systolic and diastolic diameters	21.49 ± 2.83	22.24 ± 2.61	**0.02**
Indexed mean systolic and diastolic diameters	11.06 ± 1.51	11.15 ± 1.39	0.44
*Descending aorta at pulmonary bifurcation*			
Mean of systolic and diastolic diameters	23.37 ± 3.02	24.47 ± 2.68	**0.005**
Indexed mean systolic and diastolic diameters	12.20 ± 1.64	12.27 ± 1.41	0.60

Bold mean significant values that is less than or equal 0.05

### Aortic diameters and diabetes mellitus

Comparing ascending aorta diameters at its different levels in diabetic and non-diabetic patients (Table 19) showed that there is no statistically significant difference between both groups.

**Table 19:** Comparison of ascending thoracic aorta diameters in diabetic and non-diabetic groups

Variable diameter (mm)	Non-diabetics	Diabetics	*P* value
	Mean ± SD	Mean ± SD	
*Annulus*			
(Mean)	23.11 ± 2.56	23.01 ± 2.53	0.739
(BSA-indexed)	11.72 ± 1.41	11.63 ± 1.31	0.632
*Sinus*			
(Mean)	33.76 ± 3.98	33.72 ± 3.76	0.940
(BSA-indexed)	17.12 ± 2.14	17.04 ± 1.98	0.655
*STJ*			
(Mean)	26.15 ± 3.08	26.10 ± 2.97	0.798
(BSA-indexed)	13.26 ± 1.66	13.20 ± 1.64	0.458
*Tubular*			
(Mean)	30.93 ± 4.29	31.10 ± 3.73	0.366
(BSA-indexed)	15.69 ± 2.28	15.76 ± 2.32	0.773

Comparing descending aorta diameters at levels of aortic bifurcation and diaphragm in diabetic and non-diabetic patients (Table 20) showed no statistically significant difference between both groups.

**Table 20:** Comparison of descending thoracic aorta diameters in diabetic and non-diabetic groups

Variable diameter (mm)	Non-diabetic	Diabetics	*P* value
	Mean ± SD	Mean ± SD	
*Descending aorta at the diaphragm*			
Mean of systolic and diastolic diameters	21.88 ± 2.73	22.13 ± 2.70	0.37
Indexed mean systolic and diastolic diameters	11.09 ± 1.47	11.17 ± 1.35	0.59
*Descending aorta at pulmonary bifurcation*			
Mean of systolic and diastolic diameters	24.06 ± 3.01	24.52 ± 2.63	0.12
Indexed mean systolic and diastolic diameters	12.19 ± 1.57	12.38 ± 1.30	0.23

### Aortic diameters and dyslipidemia

Comparing mean ascending aorta diameters at its annulus and sinus levels in dyslipidemic and non-dyslipidemic patients (Table 21) showed statistically significant difference between both groups. There is no statistically significant difference regarding the diameters at the level of the sino-tubular junction or the tubular part between both groups.

**Table 21:** Comparison of ascending thoracic aorta diameters in dyslipidemic and non-dyslipidemic groups

Variable diameter (mm)	No dyslipidemia	Dyslipidemia	*P* value
	Mean ± SD	Mean ± SD	
*Annulus*			
(Mean)	23.32 ± 2.55	22.93 ± 2.55	0.098
(BSA-indexed)	11.87 ± 1.39	11.59 ± 1.37	**0.035**
*Sinus*			
(Mean)	34.10 ± 3.98	33.51 ± 3.88	0.124
(BSA-indexed)	17.36 ± 2.17	16.93 ± 2.03	**0.026**
*STJ*			
(Mean)	26.24 ± 3.19	26.06 ± 2.95	0.577
(BSA-indexed)	13.36 ± 1.72	13.17 ± 1.59	0.406
*Tubular*			
(Mean)	30.64 ± 4.39	31.20 ± 3.98	0.053
(BSA-indexed)	15.59 ± 2.34	15.79 ± 2.25	0.156

Bold mean significant values that is less than or equal 0.05

Comparing descending aorta diameters at levels of aortic bifurcation or the diaphragm in dyslipidemic and non-dyslipidemic patients (Table 22) showed that there is no statistically significant difference between both groups.

**Table 22:** Comparison of descending thoracic aorta diameters in dyslipidemic and non-dyslipidemic individuals

Variable diameter (mm)	No dyslipidemia	Dyslipidemia	*P* value
	Mean ± SD	Mean ± SD	
*Descending aorta at the diaphragm*			
Mean of systolic and diastolic diameters	21.86 ± 2.81	22.99 ± 2.67	0.6
Indexed mean systolic and diastolic diameters	11.13 ± 1.52	11.11 ± 1.39	0.87
*Descending aorta at pulmonary bifurcation*			
Mean of systolic and diastolic diameters	24.26 ± 3.17	24.11 ± 2.75	0.56
Indexed mean systolic and diastolic diameters	12.34 ± 1.64	12.17 ± 1.41	0.22

### Aortic diameters and smoking

**Fig. 9:**
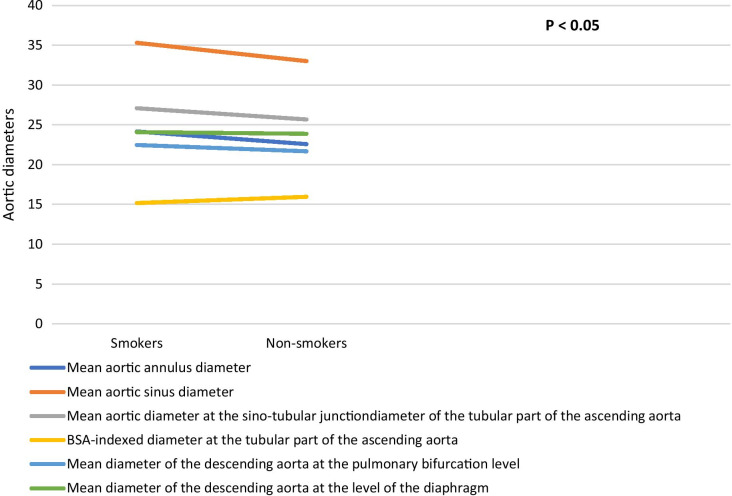
Comparison of aortic diameters at its different levels between smokers and non-smokers

Comparing ascending aorta diameters at its annulus and sinus levels in smokers and non-smokers (Table 23, Fig. 9) showed statistically significant difference between both groups. There is statistically significant difference regarding the mean diameter at the level of the sino-tubular junction between both groups. There is statistically significant difference regarding the indexed diameter at the level of the tubular part between both groups.

**Table 23:** Comparison of ascending thoracic aorta diameters in smokers and non-smokers

Variable diameter (mm)	Non-smokers	Smokers	*P* value
	Mean ± SD	Mean ± SD	
*Annulus diameter*			
(Mean)	22.58 ± 2.45	24.17 ± 2.42	**< 0.001**
(BSA-indexed)	11.61 ± 1.37	11.90 ± 1.40	**0.034**
*Sinus diameter*			
(Mean)	33.01 ± 3.88	35.31 ± 3.57	**< 0.001**
(BSA-indexed)	16.98 ± 2.14	17.37 ± 2.00	**0.022**
*STJ diameter*			
(Mean)	25.68 ± 2.89	27.09 ± 3.16	**< 0.001**
(BSA-indexed)	13.21 ± 1.63	13.32 ± 1.69	0.637
*Tubular diameter*			
(Mean)	31.01 ± 4.28	30.88 ± 3.90	0.945
(BSA-indexed)	15.96 ± 2.36	15.18 ± 2.03	**0.001**

Bold mean significant values that is less than or equal 0.05

Comparing descending aorta diameters at levels of aortic bifurcation or the diaphragm in dyslipidemic and non-dyslipidemic patients (Table 24, Fig. 9) showed that there is statistically significant difference between both groups regarding the mean diameter.

**Table 24:** Comparison of descending thoracic aorta diameters in smokers and non-smokers

Variable diameter (mm)	Non-smokers	Smokers	*P* value
	Mean ± SD	Mean ± SD	
*Descending aorta at the diaphragm*			
Mean of systolic and diastolic diameters	21.68 ± 2.63	22.47 ± 2.85	**0.002**
Indexed mean systolic and diastolic diameters	11.14 ± 1.42	11.05 ± 1.49	**0.49**
*Descending aorta at pulmonary bifurcation*			
Mean of systolic and diastolic diameters	23.88 ± 2.88	24.09 ± 2.89	**< 0.001**
Indexed mean systolic and diastolic diameters	12.24 ± 1.51	12.24 ± 1.49	0.99

Bold mean significant values that is less than or equal 0.05

### Predictors of aortic diameters

We conducted multiple linear regression analysis to find independent predictors of aortic diameters at its different levels.

### Ascending aorta (root and tubular part)

**Fig. 10:**
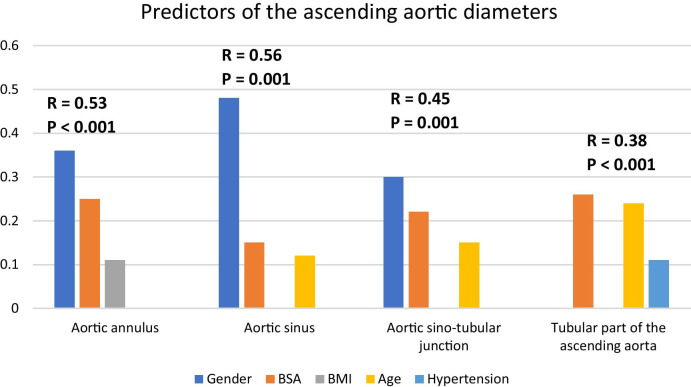
Predictors of the ascending aortic diameters at its different levels

Gender, height, and BMI were statistically significant independent predictors of aortic annular diameter as shown in Table 25, Fig. 10.

**Table 25:** Linear regression analysis to predict aortic annular diameter

Model	*R* = 0.53, adjusted *R* square = 0.28, *P* < 0.001			*P* value
	Unstandardized coefficients		Standardized coefficients	
	B	SE	Beta	
Gender, male	1.8	0.25	0.36	< 0.001
BSA	3.39	0.69	0.25	< 0.001
BMI	0.05	0.02	0.11	0.02

Table 26, Fig. 10 show that gender, BSA and age were statistically significant independent predictors of sinus diameter.

**Table 26:** Linear regression analysis to predict aortic sinus diameter

Model	*R* = 0.56, adjusted *R* square= 0.32,* P* = 0.001			*P* value
	Unstandardized coefficients		Standardized coefficients	
	B	SE	Beta	
Gender, male	3.93	0.33	0.48	< 0.001
BSA	3.16	0.83	0.15	< 0.001
Age	0.79	0.23	0.12	0.001

Table 27, Fig. 10 show that gender, BSA and age were statistically significant independent predictors of STJ diameter.

**Table 27:** Linear regression analysis to predict aortic STJ diameter

Model	*R* = 0.45, adjusted *R* square= 0.2, *P* < 0.001			Sig
	Unstandardized coefficients		Standardized coefficients	
	B	SE	Beta	
Gender, male	1.9	0.28	0.30	< 0.001
BSA	3.53	0.70	.0.22	< 0.001
Age	0.77	0.19	0.15	< 0.001

Table 28, Fig. 10 show that weight, age, BMI and hypertension were independent predictors of aortic tubular diameter.

**Table 28:** Linear regression analysis to predict aortic tubular diameter

Model	*R* = 0.38, adjusted *R* square= 0.14, *P* < 0.001			*P* value
	Unstandardized coefficients		Standardized coefficients	
	B	SE	Beta	
BSA	5.65	0.89	0.26	< 0.001
Age	1.63	0.28	0.24	< 0.001
Hypertension	0.92	0.36	0.11	0.01

### Descending thoracic aorta

**Fig. 11:**
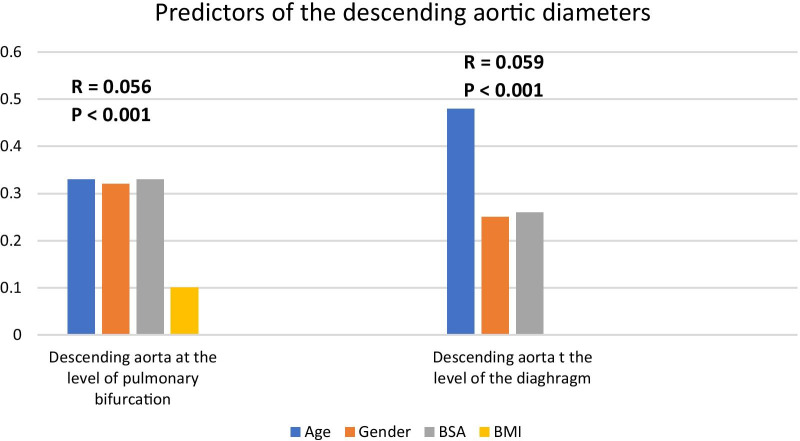
Predictors of the descending aortic diameters

Table 29, Fig. 11 show that age, gender and weight were the statistically significant predictors of mean descending aortic diameter at pulmonary bifurcation level.

**Table 29:** Linear regression analysis to predict mean aortic diameter at pulmonary bifurcation

Model	*R* = 0.56, adjusted *R* square= 0.31, *P* < 0.001			*P* value
	Unstandardized coefficients		Standardized coefficients	
	B	SE	Beta	
BSA	3.56	0.78	0.33	< 0.001
Age	1.60	0.17	0.33	< 0.001
Gender, male	1.94	0.28	0.32	< 0.001
BMI	0.05	0.02	0.10	< 0.025

Ag, BSA and gender were statistically significant independent predictors of mean aortic descending diameter at the diaphragmatic Level as shown in Table 30, Fig. 11.

**Table 30:** Linear regression analysis to predict mean aortic diameter at diaphragmatic level

Model	*R* = 0.59, adjusted *R* square= 0.35, *P* < 0.001			*P* value
	Unstandardized coefficients		Standardized coefficients	
	B	SE	Beta	
Age	2.13	0.15	0.48	< 0.001
BSA	3.80	0.56	0.26	< 0.001
Gender, male	1.39	0.22	0.25	< 0.001

## Discussion

Screening and detection of asymptomatic aortic aneurysms is based largely on uniform cut-point diameters; therefore, establishing a normal reference value for aortic diameters is important. Despite the advances in aortic imaging, there are still fewer studies covering this area of research worldwide. So, we aimed in this study to establish a normal reference value for aortic diameters among a chosen sample of the Egyptian population using MDCT. Different diameters in all thoracic aorta segments were measured. This study also shows the effect of age, gender, weight and BSA and other cardiovascular risk factors on those diameters.

### Aortic diameters

In our study, thoracic aortic diameters at its different levels (hinge to hinge measurements) were taken during systolic (40%) and diastolic (75%) phases of the cardiac cycle of contrast enhanced MSCT study. The aortic root was measured at the levels of the annulus, the sinuses of Valsalva and the STJ. The aortic diameters at the mid-level of ascending thoracic aorta and the descending thoracic aorta and at the level of pulmonary bifurcation and the diaphragm were measured. The arithmetic mean diameters (taken from systolic and diastolic diameters) and BSA-indexed diameters were calculated.

In our study, aortic root measurements were as follows**:** annulus mean diameter was 23.09 ± 2.55 mm, and its BSA-indexed diameter was 11.70 ± 1.39, sinus mean diameter was 33.75 ± 3.93 mm and its BSA-indexed diameter was 17.10 ± 2.1, STJ mean diameter was 26.13 ± 3.05 mm, and its BSA-indexed diameter was 13.25 ± 1.65. Ascending aorta mean diameter was 30.97 ± 4.16 mm and its BSA-indexed diameter was 15.71 ± 2.28. Descending aorta mean dimeter at the level of pulmonary bifurcation was 24.17 ± 2.92 mm, and its BSA-indexed diameter was 12.24 ± 1.52. Descending aorta mean dimeter at the diaphragmatic level was 21.94 ± 2.73 mm, and BSA-indexed diameter was 11.11 ± 1.44.

In *Sang Hawn Lee *et al*.* study, aortic diameters were: 29.9 ± 5.7 cm at the ascending aorta (at the middle level of the right main pulmonary artery), 23.6 ± 3.5 cm at the proximal DTA (at the middle level of the left main pulmonary artery), 21.7 ± 0.38 cm at the distal DTA (at the top of the diaphragmatic level) [6]. Ascending and descending aortic diameters were smaller by ~ 1 mm in this study than in ours. Although they measured the aorta at 9 levels, they didn’t measure any of the aortic root components and they used data from non-gated helical CT scans which could affect the accuracy of the measurements. Different ethnicity also may explain the difference.

In *Michael H.C. Pham *et al*.* study, seven anatomical segments were measured “inner edge to inner edge” using contrast-enhanced ECG-gated cardiac CTA during phase 75%. The aortic diameters were; sinus of Valsalva 33 ± 3 mm in men, 29 ± 2.5 mm in women, STJ 31 ± 3 mm in men, 27 ± 2.7 mm in women, ascending aorta at pulmonary trunk level (33 ± 4 mm in men, 30 ± 3.5 mm in women, descending aorta at pulmonary trunk level 25 ± 2 in men, 22 ± 2 mm in women, and aorta at diaphragm 23 ± 2.5 mm in men, 21 ± 2 mm in women [7]. The sinus diameters were larger in our study by ~ 2 mm, while the rest of the diameters were larger in Danish population more so at the STJ level ≥ 3 mm difference. This study is limited by including only Caucasians older than 40, and their results may, therefore, not apply in younger and non-Caucasian individuals. Furthermore, CTA images acquired at 75% of R-R interval in thoracic measures where we took our measurement both at 75% and 40% phases of the cardiac cycle.

*Barbara L. McComb *et al*.* study analyzed ungated, low-dose non-contrast CT scans. Measurements were taken outer wall to outer wall at five aortic levels; STJ, mid-ascending aorta, aortic arch, mid-descending aorta (at same level as the STJ measurements) and distal descending aorta at the diaphragmatic hiatus. Aortic diameters were: STJ 3.28 ± 0.38 cm, ascending aorta 3.38 ± 0.38 cm, mid-descending aorta 2.59 ± 0.29 cm, diaphragmatic hiatus 2.53 ± 0.28 cm [8]. Diameters were larger here than in our study, more noticeably STJ diameter ≥ 5 mm difference. This study did not include younger participants (participants were older than 55 y) and did not measure sinus and annulus diameters so we couldn’t compare those to our measurements. They also used data from non-gated CT, which results in motion artefacts and could affect the accuracy of the measurements.

In *Fay Y. Lin *et al*.* study, aortic measurements were taken during end systolic and end diastolic phases. Short-axis aortic root measurements were made at the sinuses of Valsalva in end diastolic phase. Short-axis anteroposterior and lateral end diastolic diameters of the ascending and descending thoracic aorta were measured at the level of the main pulmonary artery bifurcation. Ascending and descending thoracic aortic measurements were made at end systolic phase in a subset of 80 patients. Axial measurements were made at end systolic phase of anteroposterior and lateral axis of the ascending and descending thoracic aorta at the level of the pulmonary artery bifurcation in a subset of 36 patients. Aortic diameters were: aortic root (sinus) 3.1 ± 0.3 cm, ascending aorta (short axis, end diastolic) 2.8 ± 0.4 cm, (short axis, end systolic) 3 ± 0.3 cm and (axial, end systolic) 3 ± 0.3 cm, descending aorta (short axis, end diastolic) 2.1 ± 0.2 cm, (short axis, end systolic) 2.2 ± 0.2 cm and (axial, end systolic) 2.3 ± 0.2 cm in overall population [9]. Diameters in this study were smaller than our study, and sinus diameter was about 3 mm smaller here. This difference could be explained because in our study we measured the mean arithmetic dimeter at systolic and diastolic phases in all patient. The rest of aortic root components and the descending aorta at diaphragmatic level were not measured here so we couldn’t completely compare with our study.

In *Alfred Hager *et al*.* study, aortic diameters were measured at seven intrathoracic levels: aortic valve sinus, ascending aorta at its maximum size, aorta just proximal the right innominate artery, proximal transverse aortic arch, distal transverse aortic arch, aortic isthmus, and aorta at the level of the diaphragmatic wall of the left ventricle. Sinus diameter 29.8 ± 4.6 mm, ascending diameter 30.9 ± 0.4.1 mm and diameter at diaphragm 24.3 ± 3.5 mm [10]. Sinus diameter was smaller in this study than ours, while descending diameter at diaphragmatic level was larger here. The rest of aortic root components and descending diameter at pulmonary bifurcation level were not measured here so we couldn’t compare.

In *Ian S. Rogers *et al*.* study, measurements of the diameters of the ascending and descending thoracic aorta were acquired at the level of the right pulmonary artery. They were traced manually from outside wall to outside wall in the anteroposterior and transverse planes. For all men, the average diameters were 34.1 ± 3.9 mm for the ascending aorta, 25.8 ± 3.0 mm for the descending thoracic aorta. For all women, the average diameters were 31.9 ± 3.5 mm for the ascending aorta and 23.1 ± 2.6 mm for the descending thoracic aorta [11]. Ascending diameters in this study were larger than in our study in both men and women; however, there was no significant difference in the descending aortic diameters. One limitation of this study is the small number of participants compared to ours. Also, the measurements here were taken during one phase only (early diastole) of the cardiac cycle.

*Arik Wolak *et al*.* study performed non-contrast gated CT, and ascending and descending thoracic aortic diameters were measured at the level of pulmonary artery bifurcation. The mean aortic diameters for the ascending and descending aorta, respectively, were 33 ± 4 mm and 24 ± 3 mm for ascending and descending aorta, respectively [12]. Ascending diameter was larger here than in our study; however, they only measured the aorta at 2 levels, so more detailed comparison to our study could not be made.

### Determinants of aortic diameters

Previous studies have shown that BSA, age and gender were major determinants of aortic diameters. *Sang Hawn Lee *et al*.* study showed that age and gender were major determinants of ascending aortic diameters in asymptomatic Korean adults. They found that men had slightly larger aortic diameters than women (*P* < 0.05). Women had slightly larger BSA-adjusted aortic diameters than men (*P* < 0.05). Women’s aortic diameters were bigger than men’s in terms of the ascending aorta, while the opposite was true for the aorta between the proximal descending thoracic aorta (*P* < 0.01), when adjusted by age, hypertension, height and weight. All aortic diameters increased with height (*P* < 0.05), and all aortic diameters increased with weight (*P* < 0.05). There was a significant increase in aortic diameter at all levels throughout adult life (*P* < 0.01). All diameters increased with hypertension when adjusted by sex, age, height and weight (*P* < 0.01). This study is limited by the use of data from non-gated helical CT scan, because ECG-gated MDCT provides high resolution images in near isotropic conditions [6]. BMI was not calculated here.

In *Michael H. C. Pham *et al*.* study, diameters were body height-adjusted. This study also showed that gender, age and body surface area were significantly associated with increasing aortic diameters at all aortic segments (*P* < 0.001). All diameters were found to be larger in men than in women, but when diameters were adjusted for body height to the power of 2.7, all aortic regions except the sinus of Valsalva were found to be marginally larger in women than in men (*P* < 0.001) [7].

*Fay Y. Lin *et al. study showed that aortic root diameter was greater in men than in women and it was associated strongly with body size and less strongly with SBP and DBP. Age and BSA were independent determinants of aortic root diameters, whereas gender was not. Age and BSA were also significantly related to end-diastolic ascending and descending thoracic aortic diameter [9].

In *Brbara L. McComb *et al*.* study, it was found that age, gender, BSA, and hypertension were significant predictors of aortic diameter_._ It also showed that the aortic diameter for the diaphragmatic hiatus might be larger in current smokers [8]. On the other hand, *Hager *et al*.* study revealed no influence of weight, height, or body surface area, but it did reveal influences of gender and age. Age being the significant influencer, as there was a significant increase of the aortic diameters at all intrathoracic levels throughout adult life [10].

*Rogers *et al*.,* found that gender, age, BSA and diastolic blood pressure were significant determinants of all thoracic aortic diameters. This study was limited by the inclusion of only Caucasian participants. Also, it was limited by the lack of use of intravenous contrast [11].

*Arik Wolak *et al*.,* found that for both the ascending and descending aorta, age, BSA, diabetes, hypertension and an interaction between age and male gender (such that older men have, on average, larger aorta than women of a similar age) were significant predictors of aortic diameter. Smoking, however, was found to be independent predictor of descending aortic diameter [12].

Our study showed that gender, age, BSA, BMI and hypertension were the major determinants of aortic diameters. Diabetes has no effect on aortic diameters at its different levels.

Gender was the most important determinant of aortic root diameters (*R* = 0.53, adjusted *R* square= 0.28, *P* < 0.001 with the highest standardized coefficients beta of 0.36 to predict the annulus diameter, *R* = 0.56, adjusted *R* square= 0.32, *P* = 0.001 with the highest standardized coefficients beta of 0.48 to predict the aortic sinus diameter, *R* = 0.45, adjusted *R* square= 0.2, *P* < 0.001 with highest standardized coefficients beta of 0.30 to predict the sino-tubular junction diameter). The effect of gender on the descending aorta is less pronounced. Mean aortic diameters were significantly larger in males, with the greatest difference at the aortic root (up to 4 mm) except BSA-indexed tubular aortic diameter that was larger in females.

Age was a major determinant of thoracic aortic diameters at all levels except the annulus. It should be noted that all diameters increased with age except the annulus diameter which appeared to be smaller in the older age groups (23.55 ± 2.54 mm, 23.20 ± 2.62 mm, 22.61 ± 2.34 mm, *P* = 0.025) at age group < 40 years, 40–60 years and > 60 years, respectively), that could be explained by our method of measurement of the oval shaped annulus which was done in a single plane and without perimeter or area derived measurements. Calcifications of the annulus at the older age group led to smaller measurements. The effect of age was most pronounced on the descending aorta at the diaphragmatic level (multiple regression analysis to predict descending aortic diameter at the diaphragmatic level; *R* = 0.59, adjusted *R* square= 0.35, *P* < 0.001 with the highest standardized coefficients beta that was 0.48).

BSA was a major determinant of thoracic aortic diameters at both the ascending and descending parts. BMI was the least important determinant of aortic diameters. It had little contribution to aortic annulus diameter and descending aorta diameter at the pulmonary bifurcation level.

Regarding other cardiovascular risk factors, smoking was associated with larger aortic root and descending aortic diameters (24.17 ± 2.42 mm, 22.58 ± 2.45 mm for smokers and non-smokers, respectively, with a *P* < 0.001 at the annulus, 35.31 ± 3.57 mm, 33.01 ± 3.88 mm for smokers and non-smokers, respectively, with a *P* < 0.001 at the aortic sinus, 27.09 ± 3.16 mm, 25.68 ± 2.89 mm for smokers and non-smokers, respectively, with a *P* < 0.001 at the aortic sino-tubular junction, 22.47 ± 2.85 mm, 21.68 ± 2.63 mm for smokers and non-smokers, respectively, with a *P* = 0.002 at the descending aorta at the diaphragm, 24.09 ± 2.89 mm, 23.88 ± 2.88 mm with a *P* < 0.001 at the level of pulmonary bifurcation). Aortic tubular BSA- indexed diameter was slightly smaller in smokers (15.18 ± 2.03 mm, 15.96 ± 2.36 mm for smokers and non-smokers, respectively, with a *P* = 0.001).

Dyslipidemia was associated with smaller aortic annulus and sinus BSA-indexed diameters (11.59 ± 1.37 mm, 11.87 ± 1.39 mm, *P* = 0.035 at the aortic annulus and 16.93 ± 2.03 mm, 17.36 ± 2.17 mm, *P* = 0.026 at the aortic sinus for patients with dyslipidemia and those with no dyslipidemia, respectively).

HTN was an important determinant of tubular aortic diameter (*R* = 0.38, adjusted *R* square= 0.14, *P* < 0.001 with a standardized coefficients beta of 0.11). Aortic tubular diameters were larger in hypertensives (31.69 ± 4.17 mm, 29.94 ± 3.94 mm, *P* < 0.001 for hypertensive and normotensive patients, respectively, and the significance is maintained when the diameters were indexed to the BSA). Descending aortic diameters were larger in hypertensives (24.47 ± 2.68 mm, 23.37 ± 3.02. *P* = 0.005 for hypertensive and normotensive patients and 22.24 ± 2.61 mm, 21.49 ± 2.83 mm, *P* = 0.005 for hypertensive and normotensive patients at the level of pulmonary artery bifurcation and the diaphragm, respectively). However, BSA-indexed annulus diameter was smaller in hypertensive group (11.49 ± 1.32 mm, 12.00 ± 1.42 mm, *P* < 0.001 for hypertensive and normotensive patients. The mean annulus diameter is smaller in hypertensive group, but statistically non-significant.

## Conclusions

Our study established a normal reference value for thoracic aortic diameters among Egyptians using contrast enhanced MSCT which is an accurate, accessible and easy non-invasive method for imaging the aorta. These will aid in the diagnosis and follow-up of different aortic diseases in acute as well as chronic presentations. The study also showed major determinants of aortic diameters at its different levels which included gender, BSA, age, BMI and hypertension.

## Data Availability

The datasets used and/or analyzed during the current study are available from the corresponding author on reasonable request.

## Abbreviations

AAS: Acute aortic syndrome
CT: Computed tomography
MRI: Magnetic resonance imaging
ECG: Electrocardiogram
MDCT: Multi-detector computed tomography
MSCT: Multi-slice computed tomography
BMI: Body mass index
BSA: Body surface area
WHO: World health organization
FOV: Field of view
Kv: Kilo volt
mA: Milliampere
STJ: Sino-tubular junction
SCCT: Society of Cardiovascular Computed Tomography
SPSS: Statistical Package of Social Science
SD: Standard deviation
